# Correction: Hepatoenteric recycling is a new disposition mechanism for orally administered phenolic drugs and phytochemicals in rats

**DOI:** 10.7554/eLife.84431

**Published:** 2022-11-02

**Authors:** Yifan Tu, Lu Wang, Yi Rong, Vincent Tam, Taijun Yin, Song Gao, Rashim Singh, Ming Hu

**Keywords:** Rat

Tu Y, Wang L, Rong Y, Tam V, Yin T, Gao S, Singh R, Hu M. 2021. Hepatoenteric recycling is a new disposition mechanism for orally administered phenolic drugs and phytochemicals in rats. *eLife* **10**:e58820. doi: 10.7554/eLife.58820.

Published 1 July 2021

Recently, we identified some calculation errors in the data reported in Table 1. The calculation errors arose when we calculated the average values and the standard deviations (SDs). The average values were presented in percentage units while the SDs remained in the original format (e.g., “0.423±0.2” was presented as “42.3 ± 0.2%”). We have checked the SDs with their corresponding average values and have now corrected the results so that the values are presented correctly. As well as correcting these calculation errors we have made several updates to the values listed in Appendix 2**—**table 5. The new tables are provided with this correction. The corrections did not change any of the conclusions of the manuscript. We sincerely apologize for the mistakes.

Table 1 after correction (corrected values highlighted for illustrative purposes in bold):

**Table 1.** Comparison of bililary secretion rates and recycle ratios of glucuronides following hepatic glucuronide infusion, hepatic aglycone infusion and small intestinal aglycone perfusion.

The rate of hepatic infusion was 20 nmol/hr and the rate of intestinal perfusion was 24 nmol/hr

**Table:** 

Dosing method	Hepatic glucuronide infusion	Hepatic aglycone infusion	Small intestine aglyconeperfusion
Infused compounds	Eze-4'-G	Ezetimibe (Eze)	Ezetimibe (Eze)
	Won-7-G	Wongonin (Won)	Wongonin (Won)
	Ral-6-G	Raloxifene (Ral)	Raloxifene (Ral)
	Api-7-G	Apigenin (Api)	Apigenin (Api)
	Bai-7-G	Baicalein (Bai)	Baicalein (Bai)
Measured compound	Bile secretion rate(nmol/hr)		
Eze-4'-G	19.31 ± 1.85	2.93 ± 0.41**^*,†^	21.94 ± 5.29
Won-7-G	16.30 ± 4.28	8.46 ± 3.93**^*,†^	17.90 ± 11.96
Ral-6-G	10.53 ± 1.51	3.91 ± 0.82**^*^	4.00 ± 0.68
Api-7-G	10.64 ± 4.49	1.22 ± 0.76**^*,†^	11.32 ± 4.08
Bai-7-G	0.69 ± 0.29	0.09 ± 0.07**^*,†^	ND^†^
Measured compound	Recycle ratio(%)		
Eze-4'-G	96.54 ± 9.23	14.65 ± 2.05**^*,†^	91.42±22.04
Won-7-G	81.5 ± 21.41	42.30 ± 20.00**^*,†^	74.58±49.83
Ral-6-G	52.64 ± 7.54	19.55 ± 4.10**^*,†^	16.67±2.83
Api-7-G	53.21 ± 22.44	6.10 ± 3.80**^*,†^	47.17±17.00
Bai-7-G	3.43 ± 1.46	0.45 ± 0.35**^*,†^	ND^†^

*Significant difference between hepatic glucuronide infusion and hepatic aglycone infusion, p<0.01

†Significant difference between hepatic aglycone infusion and small intestinal perfusion, p<0.01

‡Not determined due to below quantification limit.

Table 1 before correction was provided as reference (incorrect values highlighted for illustrative purposes in bold):

**Table 1.** Comparison of bililary secretion rates and liver recycling efficiency (%) of glucuronides following hepatic glucuronide infusion, hepatic aglycone infusion and small intestinal aglycone perfusion.

The rate of hepatic infusion was 20 nmol/hr and the rate of intestinal perfusion was 24 nmol/hr

**Table:** 

Dosing method	Hepatic glucuronide infusion	Hepatic aglycone infusion	Small intestine aglyconeperfusion
Infused compounds	Eze-4'-G	Ezetimibe (Eze)	Ezetimibe (Eze)
	Won-7-G	Wongonin (Won)	Wongonin (Won)
	Ral-6-G	Raloxifene (Ral)	Raloxifene (Ral)
	Api-7-G	Apigenin (Api)	Apigenin (Api)
	Bai-7-G	Baicalein (Bai)	Baicalein (Bai)
Measured compound	Bile secretion rate(nmol/hr)		
Eze-4'-G	19.31±1.85	2.93±0.41**^*,†^	21.94±5.29
Won-7-G	16.30±4.28	8.46±3.93**^*,†^	17.90±11.96
Ral-6-G	10.53±1.51	3.91±0.82**^*^	4.00±0.68
Api-7-G	10.64±4.49	1.22±0.76**^*,†^	11.32±4.08
Bai-7-G	0.69±0.29	0.09±0.07**^*,†^	ND^†^
Measured compound	LRE(%)		
Eze-4'-G	96.54±9.23	14.65±0.02**^*,†^	91.42±22.04
Won-7-G	81.5±21.41	42.30±0.20**^*,†^	74.58±49.83
Ral-6-G	52.64±7.54	19.55±0.04**^*,†^	16.67±2.83
Api-7-G	53.21±22.44	6.10±0.04**^*,†^	47.17±17.00
Bai-7-G	3.43±1.46	0.45±0.35**^*,†^	ND^‡^

*Significant difference between hepatic glucuronide infusion and hepatic aglycone infusion, *P*<09.01

† Significant difference between hepatic aglycone infusion and small intestinal perfusion, *P*<0.01

‡ Not determined due to below quantification limit.

Appendix 2**—**table 5 after correction (corrected values highlighted for illustrative purposes in bold):

**Appendix 2—table 5**. Biliary glucuronide secretion rate and liver recycling efficiency of 16 different glucuronides.

**Table:** 

Infused compound (10 μM)	Secretion rate (nmol/hr)*	LRE%†
Ezetimibe-4'-G	19.31±1.85	96.5±9.2
Wogonin-7-G(wogonoside)	16.3±4.28	81.5±21.4
Raloxifene-4'-G	11.48±2.32	57.4±11.6
Apigenin-7-G	10.64±4.49	53.2±22.4
Raloxifene-6-G	10.53±1.51	52.6±7.5
Chrysin-7-G	**9.91±1.92 ‡**	**49.6±9.6 ‡**
Genestein-7-G	**9.45±3.21 ‡**	**47.3±16.0 ‡**
Icaritin-7-G	7.46±0.72	37.3±3.6
Icaritin-3-G	5.35±1.12	26.8±5.6
Scuttelarin	4.34±1.18	21.7±5.9
Biochanin A-G	**3.48±0.75 ‡**	**17.5±3.7 ‡**
Luteolin-3’-G	1.33±0.34	6.7±1.7
Baicalin	0.69±0.30	3.4±1.5
Luteolin-7-glycoside	0.67±0.56	3.4±2.8
Quercetin-3-G	0.43±0.57	2.2±2.8
Ace-G	0.10±0.06	0.5±0.3

*The glucuronide secretion rate at the steady state was calculated by the linear regression of accumulated amount secreted in bile vs. time

†LRE (liver recycling efficiency) %=Excretion rate at steady state/infusion rate.

‡Data from previous published study [Zeng et al., 2016]

Appendix 2**—**table 5 before correction was provided as reference (original values highlighted for illustrative purposes in bold):

**Appendix 2—table 5.** Biliary glucuronide secretion rate and liver recycling efficiency of 16 different glucuronides.

**Table:** 

Infused compound (10 μM)	Secretion rate (nmol/hr)*	LRE%†
Ezetimibe-4'-G	19.31±1.85	96.5±9.2
Wogonin-7-G(wogonoside)	16.3±4.28	81.5±21.4
Genestein-7-G	**9.78 ‡**	**59.3 ‡**
Raloxifene-4'-G	11.48±2.32	57.4±11.6
Apigenin-7-G	10.64±4.49	53.2±22.4
Raloxifene-6-G	10.53±1.51	52.6±7.5
Chrysin-7-G	**9.86 ‡**	**49.3 ‡**
Icaritin-7-G	7.46±0.72	37.3±3.6
Biochanin A-G	**3.28 ‡**	**27.8 ‡**
Icaritin-3-G	5.35±1.12	26.8±5.6
Scuttelarin	4.34±1.18	21.7±5.9
Luteolin-3’-G	1.33±0.34	6.7±1.7
Baicalin	0.69±0.30	3.4±1.5
Luteolin-7-glycoside	0.67±0.56	3.4±2.8
Quercetin-3-G	0.43±0.57	2.2±2.8
Ace-G	0.10±0.06	0.5±0.3

*The glucuronide secretion rate at the steady state was calculated by the linear regression of accumulated amount secreted in bile vs. time

†LRE (liver recycling efficiency) %=Excretion rate at steady state/infusion rate.

‡Data from previous published study [Zeng et al., 2016]

The article has been corrected accordingly.

